# Saccade-related activity in the prefrontal cortex: its role in eye movement control and cognitive functions

**DOI:** 10.3389/fnint.2014.00054

**Published:** 2014-06-30

**Authors:** Shintaro Funahashi

**Affiliations:** Kokoro Research Center, Kyoto UniversityKyoto, Japan

**Keywords:** prefrontal cortex, saccadic eye movement, post-saccadic activity, context dependency, directional selectivity, frontal eye field

## Abstract

Prefrontal neurons exhibit saccade-related activity and pre-saccadic memory-related activity often encodes the directions of forthcoming eye movements, in line with demonstrated prefrontal contribution to flexible control of voluntary eye movements. However, many prefrontal neurons exhibit post-saccadic activity that is initiated well after the initiation of eye movement. Although post-saccadic activity has been observed in the frontal eye field, this activity is thought to be a corollary discharge from oculomotor centers, because this activity shows no directional tuning and is observed whenever the monkeys perform eye movements regardless of goal-directed or not. However, prefrontal post-saccadic activities exhibit directional tunings similar as pre-saccadic activities and show context dependency, such that post-saccadic activity is observed only when monkeys perform goal-directed saccades. Context-dependency of prefrontal post-saccadic activity suggests that this activity is not a result of corollary signals from oculomotor centers, but contributes to other functions of the prefrontal cortex. One function might be the termination of memory-related activity after a behavioral response is done. This is supported by the observation that the termination of memory-related activity coincides with the initiation of post-saccadic activity in population analyses of prefrontal activities. The termination of memory-related activity at the end of the trial ensures that the subjects can prepare to receive new and updated information. Another function might be the monitoring of behavioral performance, since the termination of memory-related activity by post-saccadic activity could be associated with informing the correctness of the response and the termination of the trial. However, further studies are needed to examine the characteristics of saccade-related activities in the prefrontal cortex and their functions in eye movement control and a variety of cognitive functions.

## Introduction

It is well known that both the frontal and supplementary eye fields participate in eye movement control, since (1) electrical stimulation of these areas evokes saccadic eye movements (e.g., Bruce et al., 1985); (2) lesion of these areas causes deficits in voluntary eye movements (e.g., Latto and Cowey, 1971a,b); and (3) eye movement-related activity is observed in many neurons in these areas (e.g., Bizzi, 1967, 1968; Bizzi and Schiller, 1970; Bruce and Goldberg, 1985). However, evoked eye movements have been observed by electrical stimulation not just within these eye fields but also in a rather wide area of the lateral prefrontal cortex. In recent neurophysiological studies using non-human primates, both pre- and post-saccadic activities were observed in the lateral prefrontal cortex (Funahashi et al., 1991; Takeda and Funahashi, 2002), which is located more anterior than the frontal eye field, although a great majority of saccade-related activities were post-saccadic; i.e., saccade-related activity was initiated after the initiation of saccadic eye movement or after the termination of eye movement. Most of these post-saccadic activities exhibited directional selectivity, in that this activity was observed only when the monkey performed saccades toward a certain direction. In addition, these activities exhibited context dependency, in that this activity was observed only when the saccade was goal directed, and not during spontaneous saccades during the inter-trial interval. Even though post-saccadic activity apparently has no functional significance in eye movement control, a large number of prefrontal neurons exhibit directional and context-dependent post-saccadic activities. This suggests that these post-saccadic activities must have some functional significance while the subject performs cognitive behaviors in which the prefrontal cortex participates (e.g., working memory, attention, decision-making). This article describes the characteristics of post-saccadic activity observed in the prefrontal cortex and considers possible functions of this activity in relation to the functions of the prefrontal cortex. The classification of pre- and post-saccadic activity and detailed analysis of saccade-related activities were performed in primate neurophysiological studies. Therefore, I focused on these primate physiological studies in this article.

## Historical perspective on the prefrontal contribution to eye movement control

### Effects of electrical stimulation in the frontal lobe

Ferrier (1875) used monkeys and reported that the frontal lobe participates in eye movement control. He applied electrical stimulation to part of the monkey’s frontal lobe and observed eye movements that were directed toward the side opposite the stimulated hemisphere. He reported his observations in Philosophical Transactions (Ferrier, 1875), as follows:

“It has already been stated that the antero-frontal regions of the hemispheres give no response to electrical stimulation. Only one exception to this statement is to be made, viz. that in one case irritation of these regions caused the eyes to be tuned to one or other side, according as the electrodes were placed on the opposite hemisphere (p. 433).”

Subsequently, evidence that electrical stimulation of the frontal lobe, especially of the frontal eye fields, produced eye movements has been observed in other species such as cats, dogs, and apes (chimpanzee and orang-utan) (see Crosby et al., 1952 for details). In humans, Holmes (1938) reported that a patient with damage to the frontal lobe was unable to move his eyes in response to a command or to look at an object in any direction. He concluded “the frontal centers make possible the tuning of gaze in any desired direction and the exploration of space, but they also keep under control, or inhibit, reflexes that are not appropriate to our conduct or our reactions to the world around us”. It had been known that the cortical area within which electrical stimulation produced hand or arm movements was in the pre-central sulcus. Therefore, the frontal lobe had been known to include the cortical area that supported eye movement control separately from the area that was responsible for skeletomotor control. This notion was supported by both animal and human studies. However, the size of the cortical area within which electrical stimulation could effectively evoke eye movements and the types of evoked eye movements were different among species and among investigators (Crosby et al., 1952). These differences could be due to several experimental factors. For example, since animals were anesthetized in these experiments, the animal’s state of alertness or degree of anesthesia could affect eye movements evoked by electrical stimulation. In addition, the stimulus intensity used in these experiments was not always reported. Therefore, the stimulus intensity may have been different between these studies. Further, eye movements were not recorded and analyzed in detail. These differences in experimental conditions may have resulted in the inconsistent results regarding the characteristics of eye movements evoked by electrical stimulation of the frontal lobe.

**Figure 1:**
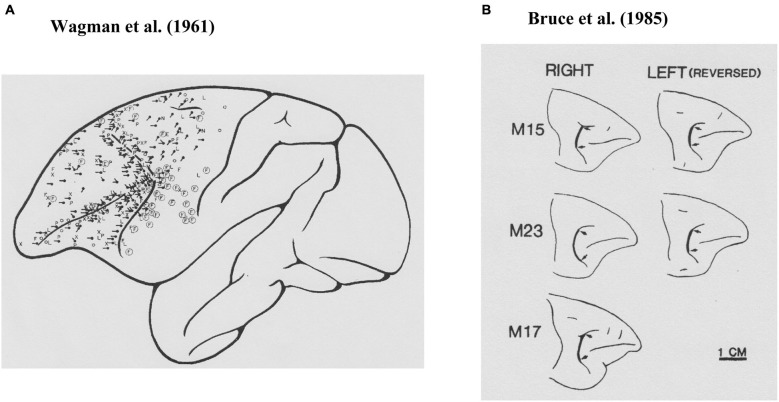
**Prefrontal cortical area where electrical stimulations evoked saccadic eye movements**. **(A)** Results from Wagman et al. (1961). The locations of the stimulation are shown as black dots and the directions of the evoked saccade are shown as black lines. **(B)** Results from Bruce et al. (1985). Their stimulation sites were localized at the thick black lines between two arrows.

Krieger et al. (1958) examined the effects of anesthesia on electrically evoked eye movements and found that the state of consciousness was important for determining the patterns of electrically evoked eye movements, as were the location stimulated and the parameters of the stimulation. Therefore, to exclude the effects of anesthesia, Wagman et al. (1961) used rhesus monkeys with high cervical transection and examined eye movements evoked by electrical stimulation of the frontal lobe. As shown in Figure 1A, they found that electrical stimulation had effects over a rather wide area of the frontal lobe. Eye movements were evoked by electrical stimulation of the cortex near the arcuate sulcus as well as the principal sulcus. The most commonly observed eye movements they found were horizontal conjugate, contralateral saccades. They also frequently observed oblique, either upward or downward, contralateral saccades. They examined the characteristics of evoked eye movements by stimulating not only the surface of the frontal lobe but also within the cortex. They also observed contralateral conjugate saccades by stimulation of the subsurface cortex. Based on these observations, they concluded that the frontal eye field extended anteriorly to the rostral limit of the principal sulcus, medially to the medial surface, and laterally to the lower limit of the arcuate sulcus.

While Wagman et al. (1961) examined electrically evoked eye movements, they neither stated parameters of electrical stimulation, nor recorded evoked eye movements. Robinson and Fuchs (1969) developed a method for accurately recording eye movements of awake monkeys and found that brief electrical stimulation (30-ms pulse train, 1-ms pulse at 200 Hz, 0.1–0.5 mA) of the frontal eye field (Brodmann’s area 8) produced a single contralateral saccadic eye movement with a typical latency of 25 ms after stimulation onset. Most of the evoked saccades were single contralateral saccades. They found that electrical stimulation of the frontal eye field produced only saccadic eye movements, and did not produce smooth pursuit, vergence, or centering eye movements or nystagmus, although these eye movements were often observed in anesthetized animals and were later found in electrophysiological studies.

Electrical stimulation of the frontal eye field consistently produced saccadic eye movements, indicating that the frontal eye field acts as a premotor region for saccadic eye movements. However, the effects of frontal eye field lesion in monkeys did not directly support this notion. For example, Latto and Cowey (1971a,b) showed that, although lesion of the frontal eye field in monkeys produced temporary visual neglect in the contralateral visual field, recovery from neglect was rapid and there was little effect on saccadic eye movements. Schiller et al. (1980) also showed that both unilateral and bilateral lesions of the frontal eye field produced only temporary deficits in eye movements. Monkeys with these lesions exhibited neglect of the contralateral visual field and made fewer saccades toward targets in that field. These results suggested that the frontal eye field participated in the initiation of visually guided saccadic eye movements. However, they showed that paired lesions of both the frontal eye field and the superior colliculus produced severe deficits in eye movements. These monkeys could no longer perform saccadic eye movements toward visual targets and the deficits showed little recovery with time. In summary, although both electrical stimulation studies and lesion studies suggested that the frontal eye field apparently participated in the initiation of visually guided saccadic eye movements, further neurophysiological studies were needed to show the timing of the discharge of neurons in the frontal eye field during saccadic eye movements.

### Saccade-related single-neuron activity in the frontal eye field

Evarts (1966) developed a method for recording single-neuron activity from the motor cortex of awake behaving monkeys. Using this method, Bizzi (1967) recorded single-neuron activities in the frontal eye field (area 8) of awake monkeys and found that the majority of frontal eye field neurons discharged in relation to eye position and that the neurons in this area discharged only after the initiation of eye movements. Subsequently, Bizzi (1968) found two types of neural activity (Type I and Type II) in the frontal eye field of monkeys. Type I neurons exhibited burst activity only after the initiation of saccadic eye movement, and this activity lasted for a very short time at the beginning of fixation. These neurons also exhibited burst activity in the fast phase of nystagmus. In addition, this burst activity was observed in complete darkness. On the other hand, Type II neurons exhibited a steady discharge when the eyes were oriented in a specific direction and were silent during saccadic eye movements. These neurons were also active during smooth pursuit eye movement and during the slow phase of nystagmus. All of these experiments were performed with the monkey’s head immobilized. Bizzi and Schiller (1970) found that the characteristic discharge patterns of Type I and Type II neurons in the frontal eye field neurons were maintained regardless of head movements.

Neurophysiological studies using awake behaving monkeys revealed that neurons in the frontal eye field (area 8) exhibit activation in relation to eye movements. Distinct groups of frontal eye field neurons participate in either saccadic eye movements or smooth pursuit eye movements. Eye movement-related activity was observed in complete darkness. These results supported the notion that the frontal eye field participates in eye movement control. However, these neurophysiological studies also showed that none of the neurons exhibited activation before the initiation of eye movement. Therefore, it was suggested that these neurons participated in some complex coordinate system used for orienting, since lesions of the prefrontal cortex had been shown to exhibit characteristic difficulties of visuomotor performances, such as difficulty in orienting vertical direction under tilted body conditions (Teuber, 1964).

Although none of the neurons in the frontal eye field exhibited responses that were exclusively related to saccadic eye movements, visually responsive neurons had been observed in the frontal eye field (Mohler et al., 1973) and were shown to exhibit enhanced visual responses when the visual stimulus was presented as the target for the saccadic eye movement (Wurtz and Mohler, 1976). Up to that point, this enhancement of visual responses was the only identified pre-saccadic activity observed in the frontal eye field in relation to eye movement. Since enhancement of the visual response was observed only when the visual stimulus was presented as the target of saccadic eye movement, this enhancement was considered to be a pre-eye movement signal and was exclusively related to eye movement. Goldberg and Bushnell (1981) further examined pre-saccadic enhancement of the visual response under a variety of task conditions and found that enhancement of the visual response was specifically observed only when the presentation of the visual stimulus preceded eye movement. Enhancement of the visual response was not observed when the visual stimulus presented within the visual receptive field was not the target of eye movement. In addition, enhancement of the visual response was not observed when the visual stimulus was the target of reaching behavior without movement of the eyes. Based on these observations, they concluded that pre-saccadic enhancement of the visual responses could be a cortical component of the neural events that preceded purposeful visually-guided saccades. Although pre-saccadic activity was found in the frontal eye field, this activity was still related to presentation of the visual stimulus and was not directly related to the control of eye movement.

Bruce and Goldberg (1985) first reported pre-saccadic activity that was directly related to saccadic eye movement in the frontal eye field. They strictly defined the location of the frontal eye field by intra-cortical micro-stimulation (Figure 1B; Bruce et al., 1985) and found that over half of the sampled neurons recorded from this area exhibited pre-saccadic activity (Bruce and Goldberg, 1985). These pre-saccadic neurons were classified into three groups (*visual*, *movement*, and *visuomovement* neurons). Among these, movement and visuomovement neurons exhibited saccade-related activity without visual stimuli. Therefore, these neurons must be directly related to eye movement control. Interestingly, movement neurons exhibited significant pre-saccadic activity only during performance of the learned saccade tasks. These neurons exhibited either no activity or significantly weaker and less consistent activity before spontaneous saccades in the dark. In addition, each neuron that showed pre-saccadic activity exhibited a broad tuning with respect to saccade direction and had an optimal direction in which the magnitude of pre-saccadic activity was maximal. The neurons that exhibited pre-saccadic activity were mostly observed within the cortical area where a low (<50 micro A) threshold electrical current evoked saccadic eye movements (Bruce et al., 1985). The directions of saccades evoked by intra-cortical micro-stimulation were usually the same as the optimal direction of pre-saccadic activity at a given recording site.

They also observed post-saccadic neurons in the frontal eye field. These neurons exhibited only post-saccadic activity following saccades made in conjunction with the tasks as well as spontaneous saccades made outside the tasks (Bruce and Goldberg, 1985). In addition, neurons that exhibited only post-saccadic activity were observed in the cortical area where higher (>100 micro A) threshold current was required to evoke saccadic eye movements. The directions of evoked saccades were different than the optimal direction of post-saccadic activity (Bruce et al., 1985).

It has been known that some frontal eye field neurons exhibited both pre- and post-saccadic activities (Bruce and Goldberg, 1985). In these neurons, both pre- and post-saccadic activity exhibited directional tuning and the optimal direction of pre-saccadic activity was usually opposite of the optimal direction of post-saccadic activity. In visually guided or memory-guided saccade tasks, monkeys usually made a saccade toward the central fixation target just after making a saccade toward the peripheral target to get a reward. Therefore, post-saccadic activity observed in these neurons was not pure post-saccadic activity but could be pre-saccadic activity related to the eye movement toward the central fixation target. This suggests that neurons exhibiting both pre- and post-saccadic activities are distinct from neurons that exhibit only post-saccadic activity.

To summarize, Bizzi (1967, 1968)initially found no neurons that exhibited pre-saccadic activity in the frontal eye field. However, an evidence that a large number of neurons with pre-saccadic activity can be found in the frontal eye field as defined by low-threshold intra-cortical micro-stimulation proved that the frontal eye field plays an important role in generating voluntary and purposive saccadic eye movements. This notion is supported by the findings that pre-saccadic activity is directionally tuned and context-dependent, such that this activity can be observed only when the subject performs a purposive eye movement and is not observed during spontaneous eye movements. On the other hand, many neurons that exhibit only post-saccadic activity are also present in the frontal eye field. This post-saccadic activity has been shown to be directionally un-tuned and not context-dependent. Therefore, neurons that exhibit pre-saccadic activity are distinct from neurons that exhibit only post-saccadic activity.

## Saccade-related activity in the dorsolateral prefrontal cortex

The cortical area that is limited to eye movement control was defined within the classical “frontal eye field” (Brodmann’s area 8; Figure 1B), which is a rather small area located at the caudal end of the principal sulcus and can be defined by whether or not saccadic eye movement is evoked by low-threshold intra-cortical micro-stimulation (<50 micro A) (Bruce et al., 1985). However, as seen in Figure 1A, electrically evoked eye movements had been observed over wide area of the lateral prefrontal cortex. Prefrontal participation in the control of motor behavior was known, since movement-related activity had been observed in the dorsolateral prefrontal cortex while monkeys performed a variety of motor tasks using the hand or arm. For example, Kubota and Niki (1971) first reported the transient excitation of prefrontal neurons when monkeys pressed a lever with their hand to get a reward in the delayed alternation task. Subsequently, Fuster (1973) observed similar movement-related activity while monkeys performed a delayed-response task with hand. Since then, activity related to behavioral responses has been widely observed when monkeys performed cognitive tasks as well as when monkeys performed simple motor tasks (see Funahashi and Takeda, 2002). Most movement-related activity exhibited selectivity, in that movement-related activity was observed only when monkeys performed a movement toward a particular direction (Kubota and Funahashi, 1982) or a certain type of behavior (e.g., go response) (Watanabe, 1986). It had been observed that movement-related activity started well before the initiation of the behavioral response (Kubota and Niki, 1971). Since the response characteristics of movement-related activity observed in the prefrontal cortex were similar to those of movement-related activities observed in the primary motor cortex (Kubota and Funahashi, 1982), this activity has been considered to participate in the initiation and execution of behavioral responses appropriate for the task.

Eye movement-related activity was also observed in the dorsolateral prefrontal cortex. Kojima (1980) used a delayed saccade task and reported saccade-related activity in the monkey prefrontal cortex. He observed a higher discharge rate during task-related saccades than during spontaneous saccades in the inter-trial interval. Joseph and Barone (1987) used a delayed oculomotor task, in which monkeys were required to perform a saccadic eye movement and an arm movement separately following visual and auditory stimuli, and examined single-neuron activity in the dorsolateral prefrontal cortex. Although they observed several types of task-related activities (e.g., signal-related pre-saccadic tonic cells, post-saccadic tonic cells, and signal-related phasic cells), they observed few saccade-related neurons, most of which exhibited post-saccadic activation. Subsequently, Barone and Joseph (1989) used a spatial sequencing task, in which monkeys were required to press three targets in the order of their illumination under either a visually guided condition or a memory-guided condition, and examined prefrontal neural activities during the monkey’s performance of this task. Although they analyzed visual tonic cells and context cells in detail, they also found that saccade-related neurons comprised 11% of their total sample (*n* = 302). They reported that, among these neurons, 85% exhibited post-saccadic activation and the remaining neurons exhibited some evidence of pre-saccadic activation. Although Joseph and Barone (1987) and Barone and Joseph (1989) used oculomotor tasks for their experiment, they mainly focused on neural activities related to cognitive functions. They did not focus on the mechanism of eye movement control by the prefrontal cortex. However, these studies showed that the dorsolateral prefrontal cortex also contained saccade-related neurons, most of which exhibited post-saccadic activation, although the proportion of saccade-related neurons was small.

Boch and Goldberg (1989) directly examined prefrontal neural activity by using oculomotor tasks to examine the characteristics of saccade-related activity (e.g., visually guided saccade task, delayed saccade task, and memory-guided saccade task). They collected single-neuron activities from the posterior third of the principal sulcal area. As with frontal eye field neurons, visual response was enhanced when the visual stimulus presented within the visual receptive field became a target of eye movement. No evoked eye movement was observed with electrical stimulation using a current as high as 150 micro A in the recording area. They observed post-saccadic activation in 11% of neurons. Post-saccadic activation had no selectivity with respect to saccade direction or amplitude and was observed during purposive saccades as well as spontaneous saccades in total darkness.

**Figure 2:**
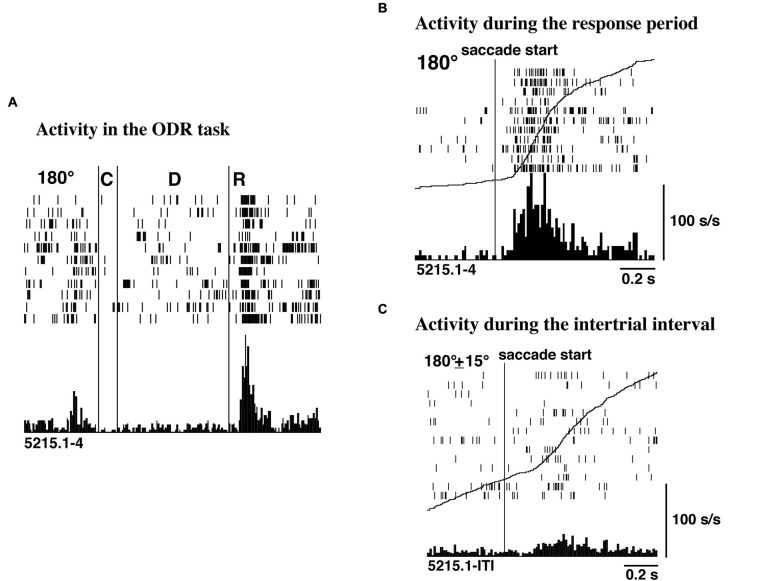
**(A)** An example of post-saccadic activity observed at the 180° trials of the ODR task. C, D, and R correspond to the cue period, the delay period, and the response period, respectively. The length of the delay period was 3 s. **(B)** Post-saccadic activity triggered at the initiation of the saccade. The activity of the same neuron as shown in **(A)**. **(C)** Activity related to spontaneous saccades occurred during the inter-trial intervals. The activity of the same neuron as shown in **(A)**. The curves drawn on **B** and **C** are not the average traces of the eye movements but the cumulative curves of neuron activities. Adapted from Funahashi et al. (1991).

**Figure 3:**
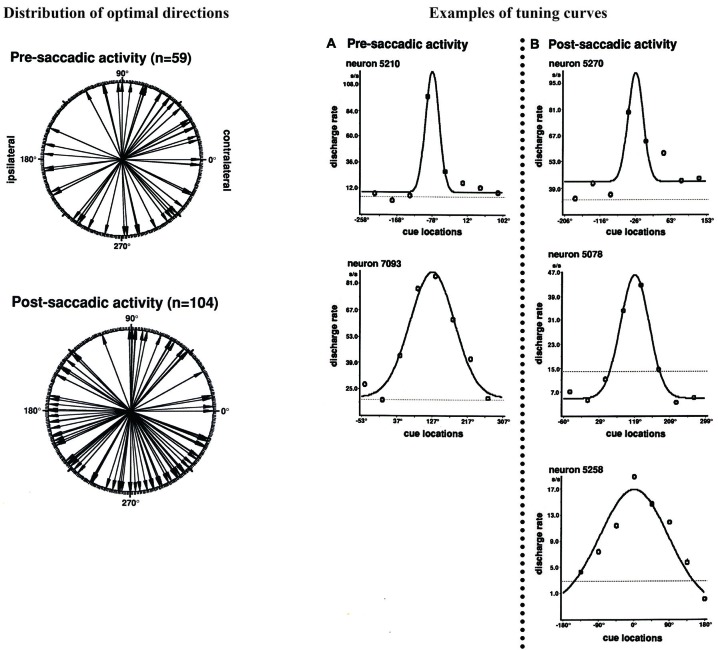
**Left**: Polar plots showing the distribution of optimal directions of pre- and post-saccadic activities. Each arrow indicates each neuron’s optimal direction of saccade-related activity, which was determined by the tuning curve. All directions were normalized as if all neural activities were collected from the left hemisphere. **Right**: Examples of tuning curves of saccade-related activities for individual neurons. The tuning curves were constructed by fitting neural activities in Gaussian function. **(A)** Pre-saccadic activity. **(B)** Post-saccadic activity. Adapted from Funahashi et al. (1991).

Funahashi et al. (1991) examined saccade-related activity of dorsolateral prefrontal neurons using a memory-guided saccade task (oculomotor delayed-response task, ODR task) and a visually guided saccade task, and found that a large number of neurons exhibited saccade-related activation in dark. Among 434 neurons recorded from the principal sulcal area, one third exhibited saccade-related responses in the ODR task. Among the neurons with saccade-related activity, a great majority (78%) exhibited post-saccadic activity and the remaining 22% exhibited pre-saccadic activity. Figure 2 shows an example of post-saccadic activity. Nearly all of the neurons that exhibited either pre-saccadic or post-saccadic activity showed directional selectivity (Figure 3). For the majority (62%) of neurons with pre-saccadic activity, the best direction was toward the visual field contralateral to the recording hemisphere, and the best direction for the remaining neurons was toward the ipsilateral field (23%) or along the vertical meridian (15%). On the other hand, 48% of neurons with post-saccadic activity had a best direction toward the contralateral visual field, and the best direction for the remaining neurons was toward the ipsilateral field (36%) or along the vertical meridian (16%). The comparison of saccade-related activity between the ODR task and visually guided saccade task revealed that most of the neurons exhibited highly similar profiles of directional selectivity and response magnitude in the two tasks. In addition, both pre- and post-saccadic activities were observed only in conjunction with task-related saccades. As shown in Figure 2, prefrontal neurons exhibited post-saccadic activity only when the monkey performed purposive saccades leading to a reward. Comparable activity was not observed in association with saccades performed during the inter-trial interval in dark. This context dependency of post-saccadic activity is one of the important features of prefrontal neurons.

**Figure 4:**
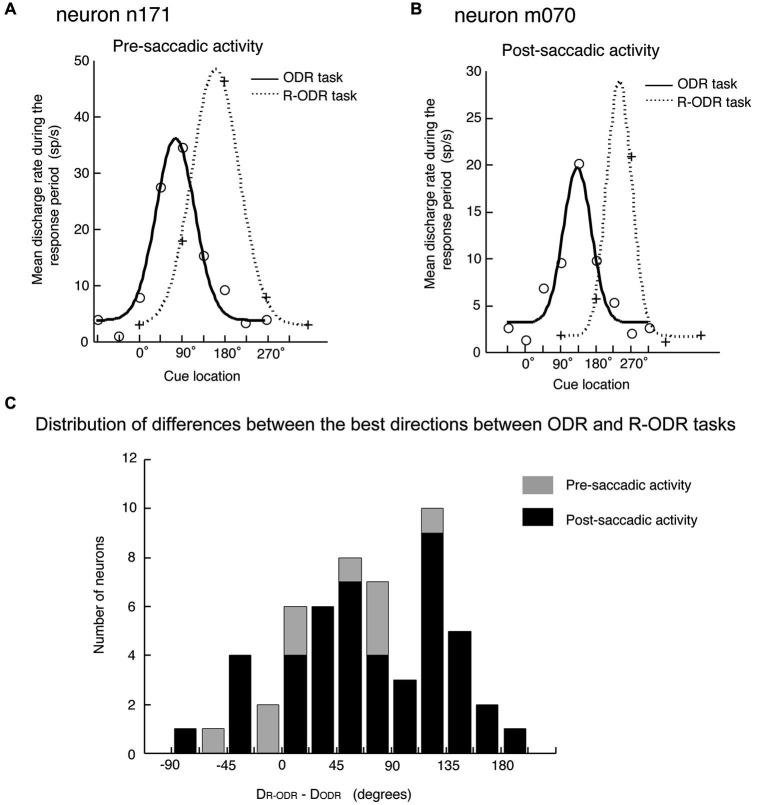
**Comparison of the directional tuning of saccade-related activities between the ODR and R-ODR tasks**. **(A)** An example of pre-saccadic activity. **(B)** An example of post-saccadic activity. **(C)** Distribution of differences between the best directions in the ODR and R-ODR tasks. The direction of the saccade was the same as the direction of the visual cue in the ODR task, but 90° clockwise from the direction of the visual cue in the R-ODR task. Therefore, if the best direction of saccade-related activity was the same in these two tasks, we could conclude that this activity encoded the direction of the visual cue. On the other hand, if the best direction of saccade-related activity in the R-ODR task was 90^o^ clockwise from the best direction in the ODR task, we could conclude that this activity encoded the direction of the saccade. Adapted from Funahashi and Takeda (2002).

The original observation by Funahashi et al. (1991) was confirmed by Takeda and Funahashi (2002), who examined information represented in pre- and post-saccadic activities using two oculomotor tasks; the ODR task and the rotatory ODR (R-ODR) task. The direction of the saccade was the same as the direction of the visual cue in the ODR task but 90° clockwise from the direction of the visual cue in the R-ODR task. By comparing the directional tuning of saccade-related activity in the ODR and R-ODR conditions, we could determine whether each saccade-related activity encoded the direction of the visual cue or the direction of the saccade. For example, if the preferred direction of saccade-related activity was the same between these two tasks, we could conclude that this activity encoded the direction of the visual cue. On the other hand, if the preferred direction of saccade-related activity in the R-ODR task was 90° clockwise from the preferred direction in the ODR task, we could conclude that this activity encoded the direction of the saccade. Takeda and Funahashi (2002) showed that, among 57 neurons that exhibited directional activity during the response period, 58% encoded the direction of the saccade and 35% encoded the direction of the visual cue (Figure 4). Both pre- and post-saccadic neurons were included in the latter group. Interestingly, most of the neurons with saccade-related activity that encoded the direction of the visual cue also exhibited directional delay-period activity which encoded the direction of the visual cue, and the preferred directions of both activities were almost identical. These results indicate that not all saccade-related activities are related to the execution or control of saccadic eye movement. Although the activity is generated in relation to eye movement, some saccade-related activities of prefrotal neurons apparently participate in functions other than eye movement control.

In summary, it has been shown that the dorsolateral prefrontal cortex includes a large number of saccade-related neurons. Although most saccade-related activity was post-saccadic, a significant proportion of saccade-related neurons exhibit pre-saccadic activity. The characteristics of directional selectivity and context dependency are similar between pre- and post-saccadic activities in the prefrontal cortex. These results indicate that the prefrontal cortex participates in the control of purposive saccadic eye movements, similar to the frontal eye field. However, since most saccade-related activity in the prefrontal cortex was post-saccadic and since some saccade-related activities of prefrontal neurons apparently participate in functions other than eye movement control, the prefrontal cortex and the frontal eye field may make different contributions to the control of saccadic eye movements.

## Comparison of saccade-related activity between the prefrontal cortex and the frontal eye field

An important feature of the saccade-related activity of prefrontal neurons is that both pre- and post-saccadic activities are context-dependent, i.e., prefrontal neurons exhibited saccade-related activity only when the monkey performed purposive saccades (Funahashi et al., 1991). Both pre- and post-saccadic activities were either greatly reduced or absent during spontaneous saccades. However, neurons in the frontal eye field exhibit context dependency only for pre-saccadic activities, and not for post-saccadic activity (Bruce and Goldberg, 1985). Context-dependency only for pre-saccadic activity had also been shown in other brain areas including the posterior parietal cortex (area 7a) (Lynch et al., 1977), the caudate nucleus (Hikosaka et al., 1989), and the substantia nigra pars reticulata (Hikosaka and Wurtz, 1983). In these brain areas, most neurons exhibited pre-saccadic activity during goal-directed saccades, but only weak or no activity during spontaneous saccades with the same direction and magnitude in the dark.

Another important feature of saccade-related activity in the prefrontal cortex is that most neurons exhibited only post-saccadic activity (Funahashi et al., 1991). Post-saccadic activity began at the same time of the initiation or well after the termination of saccadic eye movements. The median latency of post-saccadic activity was 122 ms after the initiation of the eye movement. The mean duration of saccadic eye movements was 45 ms (Funahashi et al., 1989). Therefore, most post-saccadic activity in the prefrontal cortex initiated more than 50 ms after the completion of saccadic eye movements in the dark.

Although post-saccadic activity is neglected in many reports because this activity does not have any clear role in the initiation and control of saccadic eye movements, post-saccadic activity has usually been considered to be an evidence of a corollary discharge from structures that are directly responsible for eye movement. If post-saccadic activity is a corollary discharge from the oculomotor centers, then it should be observed with any saccade regardless of whether it is purposive or spontaneous. In fact, neurons in the oculomotor centers, such as the brainstem premotor nuclei (Hepp et al., 1989) and the superior colliculus (Sparks and Mays, 1980), were active during every saccade regardless of whether it was rewarded or spontaneous. In fact, post-saccadic activity in the inferior and lateral pulvinar (Robinson et al., 1986) and the frontal eye field (Bizzi, 1968; Bizzi and Schiller, 1970; Bruce and Goldberg, 1985; Bruce et al., 1985) was observed during saccades regardless of whether it was rewarded or spontaneous. Therefore, post-saccadic activity in these structures is likely to be a corollary discharge. On the other hand, post-saccadic activity observed in the prefrontal cortex was context-dependent, in that post-saccadic activity was observed only during saccades in relation to the reward and became significantly weak or was not observed during spontaneous saccades. Therefore, post-saccadic activity observed in the prefrontal cortex is not a corollary discharge from oculomotor structures in the brainstem.

In addition, in the frontal eye field, directional preference and contralateral bias were observed for pre-saccadic activity, but not for post-saccadic activity (Bruce and Goldberg, 1985). However, in the prefrontal cortex, most pre- and post-saccadic activities exhibited directional preference and contralateral bias, although the contralateral bias for pre-saccadic activity was stronger than that for post-saccadic activity (Figure 3; Funahashi et al., 1991).

In summary, the prefrontal cortex includes a large number of saccade-related neurons, most of which exhibit post-saccadic activity. Although post-saccadic activity is initiated well after the initiation of saccadic eye movement, this activity is clearly related to saccadic eye movement, since it exhibits selectivity with respect to the saccade direction and directional tuning. However, in contrast to the traditional idea that post-saccadic activity is a corollary discharge from oculomotor centers, post-saccadic activity observed in the prefrontal cortex is not a corollary discharge because of its context-dependent nature. These results indicate that post-saccadic activity plays some specific roles in cognitive functions in which the prefrontal cortex participates.

## Contribution of post-saccadic activity to cognitive functions

### Post-trial activities observed in the prefrontal cortex

Post-saccadic activity is initiated after the initiation of saccadic eye movement. Some neurons with post-saccadic activity start firing well after the termination of saccadic eye movement. Several post-trial activities have been found in the prefrontal cortex, and post-saccadic activity could be classified as one of these. For example, Rosenkilde et al. (1981) reported three types of post-trial activities while monkeys performed delayed-response and delayed matching-to-sample tasks. The post-trial activities that they observed depended on the presence or absence of the reward. One type of post-trial activity (Type I cells) was related to reward delivery. This activity was observed after correct, reinforced responses regardless of the task conditions. This activity was also observed after the delivery of free reward without any response. No activity was observed after unreinforced responses regardless of whether the response was correct or not. Therefore, this activity is considered to be directly related to reward delivery. Another type of post-trial activity (Type II cells) was not observed after correct, reinforced responses, but was observed in extiction trials with correct but unreinforced responses. They stated that this type of activity encoded deviations from expectancy of the reward. The third type of post-trial activity (Type III cells) was observed after all responses regardless of the presence or absence of the reward. They concluded that this activity encoded termination of a trial.

Watanabe (1989) further characterized post-trial activities observed in the prefrontal cortex. In addition to neurons (*juice-related units* and *end-of-trial-related units*) with responses similar to those of the Type I and III cells described by Rosenkilde et al. (1981), several other kinds of neurons that exhibit post-trial activities have been reported. For example, *reinforcement-related units* responded only when the reward was given for the correct response and did not respond to the free reward. *Reinforcement-error-related units* responded after both correct and error responses, but the responses in each case were different. He found some reinforcement- and reinforcement-error-related units that encoded the correctness of the response, since the responses of these units depended only on the correctness of the response and were independent of the presence and absence of the reward. In addition, he found two kinds of *error-related units* that responded only when the monkey made an error: one was related to error recognition and the other was related to encoding the absence of an expected reward, which is similar to the Type II cells described by Rosenkilde et al. (1981).

Neurons in the prefrontal cortex exhibit several kinds of post-trial activities. Since reward delivery is always provided at the end of a correct trial, reward-related activity (e.g., Type I cells, Type II cells, juice-related units, reinforcement-related units, and error-related units) could be observed in any type of experiment performed in the prefrontal cortex. Actually, reward-related activity was observed when the ODR task was used to examine prefrontal activities (Ichihara-Takeda and Funahashi, 2006). In the ODR task, the monkey was required to perform memory-guided saccades toward one of eight directions. The same amount of reward was delivered in every correct trial immediately after the end point of the saccade entered within the target zone. Therefore, no directional tuning should be observed in reward-related activity. As was explained before, a great majority of post-saccadic activity exhibited directional selectivity in the prefrontal cortex (Funahashi et al., 1991; Takeda and Funahashi, 2002). Therefore, post-saccadic activity is not a kind of reward-related activity. On the other hand, activity that encoded the end of the trial (Type III activity and end-of-trial-related activity) and activity that encoded the correctness of the response were observed in the prefrontal cortex when the monkeys performed cognitive tasks. Activity that encoded the end of the trial would be important for neural systems related to cognitive functions, since these systems need to prepare to receive information that is necessary for the next new trial immediately after the preceding trial is complete. Similarly, activity that encodes the correctness of the response and error-related activity that detects an error should also be important for monitoring the responses and for making flexible changes in behavior. Post-saccadic activity observed in the prefrontal cortex seems to have features similar to the activity that encodes the end of the trial and that encodes correctness, due to the context-dependent nature of post-saccadic activity.

### Post-saccadic activity could terminate delay-period activity

Funahashi et al. (1991) observed a large number of prefrontal neurons that exhibited post-saccadic activity while monkeys performed an oculomotor version of the delayed-response task (ODR task). This post-saccadic activity could be important for the prefrontal cortex to prepare to receive updated information for a new trial and to monitor responses and make flexible changes in behavior.

Using the ODR task, Funahashi et al. (1989) found that a large number of prefrontal neurons exhibited tonic, sustained activation during the delay period (delay-period activity). This delay-period activity was directionally tuned, in that it was observed only when visual cues were presented at a particular area in the visual field, usually the visual field contralateral to the recording hemisphere. Delay-period activity has several important features (Funahashi et al., 1989). First, the duration of delay-period activity is either prolonged or shortened depending on the length of the delay period. Second, this activity is observed only when monkeys perform correct behavioral responses. Third, a great majority of delay-period activity exhibits a directional preference, in that it is observed only when a visual cue is presented at a particular area in the visual field. The preferred direction of this activity differs from neuron to neuron. Therefore, it has been proposed that neurons that exhibit directional delay-period activity have mnemonic receptive fields (memory fields) in the visual field, analogous to visual receptive fields. Fourth, it has been shown that the great majority of delay-period activity represents information regarding the direction of the visual cue (retrospective information), whereas the minority represents information regarding the direction of the saccade (prospective information) (Funahashi et al., 1993; Takeda and Funahashi, 2002). Based on these observations, delay-period activity has been considered to be a neural correlate of temporary information-storage processes in working memory (Goldman-Rakic, 1987; Funahashi, 2001; Fuster, 2008).

While a monkey performs the delayed-response task, the ability to retain spatial information regarding the visual cue “on line” as directional delay-period activity is essential for the monkey to correctly perform eye movements in the response period. However, directional delay-period activity is no longer necessary once a saccadic eye movement has been made. The presence of delay-period activity may even be counterproductive if it persists into the next trial, since the subject must refresh his memory in each new trial. Funahashi et al. (1989) showed that delay-period activity was actively terminated immediately after the subject made saccadic eye movements. A great majority of post-saccadic activity exhibited directional selectivity. The distributions of the preferred directions and tuning widths were similar between post- and pre-saccadic activities in the prefrontal cortex. These results suggest that post-saccadic activity is a feedback signal from the brain areas that are responsible for the initiation of purposive saccades. Therefore, post-saccadic activation observed in the prefrontal cortex may be a neural signal from these brain areas and may play a critical role in terminating delay-period activity after the execution of eye movement.

**Figure 5:**
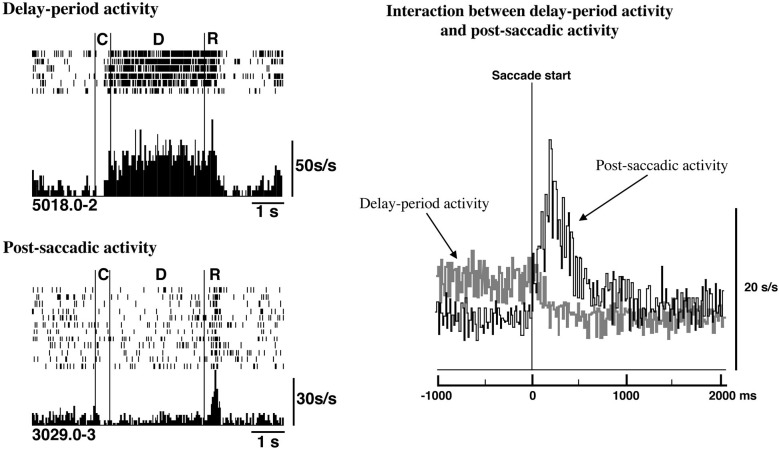
**Relations between delay-period activity and post-saccadic activity**. **Top Left**. Delay-period activity was tonically active until the eye movement began during the response period. **Bottom Left**. An example of post-saccadic activity. **Right**. Populations of delay-period activity and post-saccadic activity were aligned at the initiation of the saccade. The termination of delay-period activity coincided with the initiation of post-saccadic activity at a population level. Adapted from Goldman-Rakic et al. (1990).

In fact, as shown in Figure 5, delay-period activity terminated rapidly when saccadic eye movement was initiated. At the same time, post-saccadic activity started increasing. Thus, the termination of excitatory delay-period activity coincided with the initiation of post-saccadic activity based on population analyses of prefrontal activities (Goldman-Rakic et al., 1990; Funahashi and Takeda, 2002). This result supports the idea that post-saccadic activity could play a critical role in terminating delay-period activity after the execution of eye movement. The termination of delay-period activity at the end of the trial ensures that the subject is prepared to receive updated information. This should contribute to the flexible modulation of behavior.

### Post-saccadic activity may participate in performance monitoring

Activity that encodes the correctness of the response and activity that detects errors have been observed in the prefrontal cortex in monkeys as well as humans (see reviews by Ridderinkhof et al., 2004; Mansouri et al., 2009). These activities should be important for monitoring responses and enabling the flexible modulation of behavior. It has been proposed that the anterior cingulate cortex contributes to monitoring performance by detecting errors. For example, Carter et al. (1998) observed activation of the anterior cingulate area during erroneous responses in the continuous performance task by human fMRI studies. However, the same area was also activated during correct responses under conditions of increased response competition, in which errors were likely to occur. Similar results have also been reported by Botvinick et al. (1999) using a version of the flanker task. They observed anterior cingulate activation even in correct trials and this activation was greater in high conflict and incongruent trials than in low conflct and congruent trials. The anterior cingulate cortex does not just detect errors, it also participates in the online monitoring of performance.

Post-saccadic activity related to the detection of errors and the monitoring of conflict among processes has been observed in the supplementary eye field. Stuphorn et al. (2000) analyzed the activities of supplementary eye field neurons while monkeys performed a countermanding task and found several types of neurons that exhibited post-saccadic activities. One type of neuron was strongly activated after the initiation of the planned eye movement specifically when monkeys failed to cancel it. Although this activity can be classified as post-saccadic activity, they stated that this activity is distinct from post-saccadic activity observed in the frontal eye field because this activity was not observed in trials without the stop signal, in which monkeys made visually-guided saccades toward the target, and because this activation was observed in both ipsiversive and contraversive saccades. Based on these observations, they concluded that this activity is a signal of the occurrence of the error. A second type of neurons was also activated only when the initiation of the planned eye movement was cancelled. However, this activation was not likely to be related to the cancelling of eye movement because the activity occurred after the stop-signal reaction time, which is the timing after the initiation of eye movement in non-cancelled stop-signal trials. When eye movements are cancelled in the countermanding task, gaze-shifting neurons (saccade-related neurons) and gaze-holding neurons need to be co-activated, but this co-activation produces a conflict in processing and the balance of activation between these two groups of neurons is critical for determining whether the eye movement will be cancelled or not. Therefore, they suggested that the second type of activity signals a conflict in processing. Although post-saccade-like activity was observed in the supplementary eye field while monkeys performed a countermanding task, Stuphorn et al. (2000) concluded that these activities represented performance monitoring, such as signals that detect errors and a conflict in processing.

Prefrontal participation in flexible behavioral control and behavioral adjustment to dissolve the conflict has been examined using the anti-saccade task and categorization tasks (Munoz and Everling, 2004; Mansouri et al., 2009). Recently, Teichert et al. (2014) focused on post-saccadic activities in the frontal eye field and tried to understand these activities in more cognitive terms, such as reward expectation, decision uncertainty, or response conflict. If post-saccadic activity in the frontal eye field is related to functions such as reward expectation, decision uncertainty, or response conflict, this activity should differ between correct and error trials. To determine whether the activity in the frontal eye field reflects choice errors, they examined error-related activity while monkeys performed a reward-biased speed-categorization task with delayed feedback. Since monkeys often made impulsive errors in this task, they could analyze a sufficient amount of internally generated error-related activities in the frontal eye field. As a result, they found that error-related activity was observed in a large fraction of frontal eye field neurons and that the magnitude of error-related activity was strongly correlated with the difficulty of the task and not with eye movements. Therefore, post-saccadic activity observed in the frontal eye field might be related to the evaluation of post-decision outcome. Since the evaluation of the preceding choice could play a role in optimizing the subsequent behavior, post-saccadic activity might play a role in monitoring performance.

As stated before, activity that encodes the end of the trial and activity that encodes the correctness of the response have been observed in the prefrontal cortex while monkeys performed cognitive tasks using hand or arm movements. These activities clearly exhibit context-dependent features. In addition, error-related activity that is modulated by the difficulty of the trial also exhibits context-dependent features, as does the post-saccadic activity observed in the prefrontal cortex. Since post-saccadic activity observed in the prefrontal cortex exhibited directional selectivity and was observed only during the response period of the task, this activity might have a specific role, such as terminating delay-period activity. This function could also be considered a type of performance monitoring, since the termination of delay-period activity by post-saccadic activity would be associated with informing the correctness of the response and the termination of the trial. The termination of memory-related activity at the end of the trial ensures that the subject is prepared to receive new and updated information. Therefore, post-saccadic activity observed in the prefrontal cortex may also participate in performance monitoring.

## Conclusions

Neurophysiological studies have revealed that neurons in the lateral prefrontal cortex exhibit saccade-related activity. Although a significant proportion of saccade-related neurons exhibited pre-saccadic activity, most neurons exhibited post-saccadic activity. Saccade-related activity in the prefrontal cortex showed directional selectivity and context dependency, such that the activity was observed only when the monkey performed goal-directed saccades. The characteristics of directional selectivity and context dependency were similar between pre- and post-saccadic activities. These results indicate that the prefrontal cortex participates in the control of purposive saccadic eye movements, similar to the frontal eye field. Based on the traditional idea that post-saccadic activity is a corollary discharge from oculomotor centers, post-saccadic activity observed in the frontal eye field is a corollary discharge because it has no directional tuning and no context dependency. However, post-saccadic activity observed in the prefrontal cortex is not a corollary discharge because of its context-dependent nature. Therefore, the prefrontal cortex and the frontal eye field might contribute differently to the control of saccadic eye movements. Post-saccadic activity in the prefrontal cortex could play some specific roles in cognitive functions in which the prefrontal cortex participates. For example, the activity that encoded the end of the trial, the activity that encoded the correctness of the response, and error-related activity were observed in the prefrontal cortex when the monkeys performed cognitive tasks. These activities could be important for monitoring the responses and for making flexible changes in behavior. Post-saccadic activity observed in the prefrontal cortex seems to have features similar to these activities, due to the context-dependent nature of post-saccadic activity. In addition, post-saccadic activity might act to terminate memory-related activity after a behavioral response is done. The termination of memory-related activity at the end of the response behavior ensures that the subject can prepare to receive updated information. This idea was supported by the observation that the termination of memory-related activity coincided with the initiation of post-saccadic activity. In addition, a recent study shows that post-saccadic activity observed in the frontal eye field as well as the supplementary eye field might be related to the evaluation of post-decision outcome. The evaluation of the preceding choice could play a role in optimizing the subsequent behavior. Therefore, post-saccadic activity might play a role in monitoring performance and making flexible changes in behavior. However, further investigations are needed to examine the characteristics of saccade-related activities observed in the prefrontal cortex and their possible contributions to eye movement control and prefrontal functions.

## Conflict of interest statement

The author declares that the research was conducted in the absence of any commercial or financial relationships that could be construed as a potential conflict of interest.
